# 5-Aminoimidazole-4-carboxamide-1-beta-D-ribofuranosyl 5'-Monophosphate (AICAR), a Highly Conserved Purine Intermediate with Multiple Effects 

**DOI:** 10.3390/metabo2020292

**Published:** 2012-03-23

**Authors:** Bertrand Daignan-Fornier, Benoît Pinson

**Affiliations:** Université Bordeaux, CNRS, IBGC, UMR 5095, F-33000 Bordeaux, France; Email: benoit.pinson@ibgc.cnrs.fr (B.P.)

**Keywords:** AICAR, AMPK, metabolism, signal transduction, purine, ATIC

## Abstract

AICAR (5-Aminoimidazole-4-carboxamide-1-beta-D-ribofuranosyl 5'-monophosphate) is a natural metabolic intermediate of purine biosynthesis that is present in all organisms. In yeast, AICAR plays important regulatory roles under physiological conditions, notably through its direct interactions with transcription factors. In humans, AICAR accumulates in several metabolic diseases, but its contribution to the symptoms has not yet been elucidated. Further, AICAR has highly promising properties which have been recently revealed. Indeed, it enhances endurance of sedentary mice. In addition, it has antiproliferative effects notably by specifically inducing apoptosis of aneuploid cells. Some of the effects of AICAR are due to its ability to stimulate the AMP-activated protein kinase but some others are not. It is consequently clear that AICAR affects multiple targets although only few of them have been identified so far. This review proposes an overview of the field and suggests future directions.

## 1. Introduction

AICAR (5-Aminoimidazole-4-carboxamide-1-β-D-ribofuranosyl 5'-monophosphate), also known as ZMP (the “Z” referring to imidaZole [1]), is an intermediate in the inosine monophosphate (IMP) conserved pathway which is responsible for *de novo* purine biosynthesis in all organisms. The recent attention paid to AICAR is testified by more than one thousand publications referenced in databases such as PubMed, 90% of which having been published during the last 10 years. This massive and sustained interest for this small molecule is due to its multiple biological effects, which will be presented in this review.

## 2. Metabolism of AICAR

**Figure 1:**
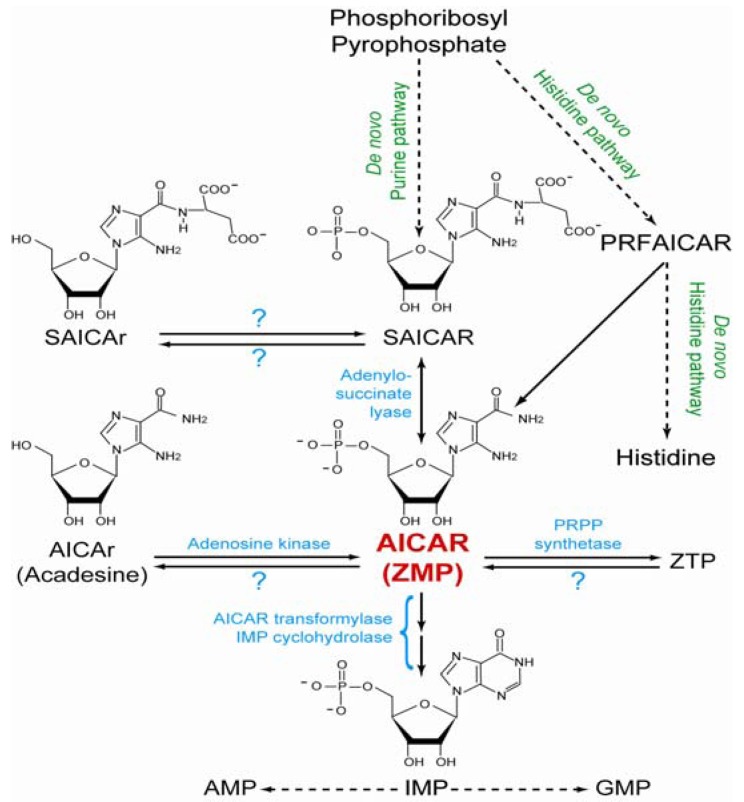
Schematic representation of the *de novo* purine and histidine pathways in yeast. AICAR: 5-Aminoimidazole-4-carboxamide-1-β-D-ribofuranosyl 5'-monophosphate. AICAr: riboside form of AICAR (also named acadesine). AMP: Adenosine 5'-monophosphate; GMP: Guanosine 5'-monophosphate; IMP: Inosine 5'-monophosphate. PRFAICAR: 5-(5-Phospho-D-ribosylaminoformimino)-1-(5-phosphoribosyl)-imidazole-4-carboxamide. SAICAR: succinyl-AICAR. SAICAr: succinyl-AICAr. ZMP: AICAR monophosphate. ZTP: AICAR triphosphate. Enzyme names are given in blue. Question marks indicate enzymatic activities catalyzed by unidentified enzymes.

AICAR is an intermediate metabolite in the purine *de novo* synthesis pathway (Figure 1), it is synthesized from succinyl-AICAR (SAICAR) by adenylosuccinate lyase (ASL), an enzyme inhibited by AICAR through a feedback regulation [2]. As a consequence, massive accumulation of AICAR is associated with SAICAR accumulation in micro-organisms such as yeast [3] and in a specific human pathology [4]. In the *de novo* purine synthesis pathway, AICAR is further metabolized to IMP by successive action of AICAR-Transformylase and IMP Cyclohydrolase, two enzymatic activities which are generally carried on a single protein named ATIC. In micro-organisms, AICAR is also synthesized as a by-product of the histidine biosynthesis pathway (Figure 1). 

Under conditions where AICAR accumulates, riboside and triphosphate derivatives are often found in cellular extracts or body fluids. A patient lacking ATIC activity showed accumulation of large amounts of AICAR riboside (also known as acadesine or AICAr) in urines and mono- di- and tri-phosphate forms of AICAR in erythrocytes [4]. The enzyme(s) dephosphorylating AICAR monophosphate to its riboside form is not identified yet, but it is clear that adenosine kinase can reverse the reaction and phosphorylate AICAR riboside to the monophosphate form [5]. Synthesis of ZTP (triphosphate form of AICAR) was found to occur directly from AICAR through the catalytic action of PRPP-synthetase [6]. Consequently, ZDP (diphosphate form of AICAR) detected in erythrocytes is likely to result from ZTP degradation and to appear upon intracellular degradation or during metabolite extraction, rather than be a ZTP synthesis intermediate. In the early eighties, ZTP was proposed to be an “alarmone” signaling folate deficiency in *Salmonella typhimurium* [1] but a later study did not confirm such a role for ZTP in *Escherichia coli* [7]. 

## 3. Roles of Physiologically Produced AICAR and Accumulation in Metabolic Diseases

**Figure 2:**
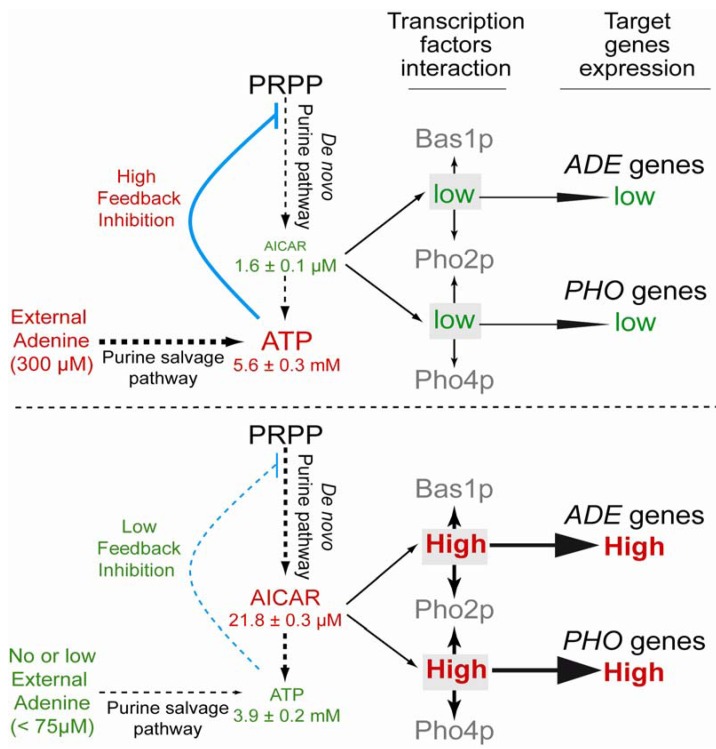
Schematic representation of AICAR physiological effects in yeast. Intracellular ATP and AICAR concentrations were determined by liquid chromatography as described in [19] on exponentially BY4741 cells grown in SD medium containing 1% casaminoacid (Difco), tryptophan (0.2 mM) and containing (upper panel) or not (lower panel) adenine (0.3 mM). *ADE* and *PHO* genes refer to Bas1-Pho2 and Pho4-Pho2 target genes, respectively.

A physiological role for AICAR has been found in yeast cells where it stimulates the interaction between two pairs of transcription factors (Bas1-Pho2 and Pho4-Pho2), thereby resulting in the transcriptional activation of specific sets of genes [3,8]. Importantly, most of the AICAR-responsive genes also respond to extracellular adenine, their expression being low when adenine is abundant in the growth medium [3,9,10,11,12,13,14]. AICAR concentration is linked to exogenous adenine through feedback regulation of the first step of the purine *de novo* pathway. This feedback regulation is thought to be mediated by ATP and ADP [2]. Consistently, in adenine replete conditions, ADP and ATP concentrations are higher [12], while AICAR concentration decreases [15]. Finally, fusion chimera between AICAR-stimulated transcription factors resulted in an adenine-independent transcriptional activation of the target genes [3,16]. These results led to a model accounting for the complex regulatory effects of AICAR in yeast and their connection to purine precursor availability in the growth medium (Figure 2). Beside these physiological effects associated to moderate AICAR accumulation, massive accumulation of AICAR can also lead to detrimental effects in yeast. Intracellular accumulation of AICAR in the millimolar range provokes histidine auxotrophy and, when combined to the *fau1* mutation affecting 5,10-methenyltetrahydrofolate synthetase, leads to methionine auxotrophy. Higher concentrations, up to 10-15 mM, result in growth arrest [15]. In yeast, physiological and detrimental effects of AICAR are only associated to its phophorylated form(s), since accumulation of the riboside form at the same concentration has no effects either on transcription, amino-acids prototrophy nor on cellular growth [15].

In mammalian cells it is not known whether endogenous AICAR plays regulatory roles. It is however striking that most purine metabolism-associated diseases result in AICAR accumulation in the patient cells [17]. The most dramatic accumulation of AICAR was observed in the erythrocytes of an ATIC-deficient patient and was associated to dysmorphic features, severe neurological defects, and congenital blindness [4]. At this point it is not clear whether some or all of these symptoms are the direct result of very high AICAR concentrations or whether they are due to the increase of AICAR derivatives and/or to the severe ATP depletion associated with AICAR massive accumulation [4]. The consequences of AICAR accumulation in other purine metabolism-associated diseases is not established, but AICAR was proposed as the possible toxic metabolite in Lesch-Nyhan disease resulting from impaired hypoxanthine-guanine phosphoribosyl transferase [18].

## 4. AICAR, a Potent Activator of AMP-Activated Protein Kinase

In the early 90s, AICAR in its monophosphate form was found to activate the human AMP-activated protein kinase (AMPK) activity *in vitro* [20]. The AICAR riboside precursor was then used to activate AMPK in isolated rat adipocytes [21]. Since then, AICAR has been used in hundreds of studies as an inducer of AMPK activity. A major advantage of AICAR compared to other AMPK-inducers is that AICAR addition at low concentration (< 500 µM) does not affect AMP, ADP or ATP levels [22]. However, more recently, effects of AICAR on ATP concentration were reported in rat hepatocytes [23,24]. This observation combined with the multiple AMPK-independent effects reported for AICAR (see below) should inspire cautious interpretation of the results (as discussed in [25]).

AICAR was found to directly interact with the gamma-subunit of AMPK. This interaction induces a conformational change and favors phosphorylation of the catalytic alpha subunit, which in turn becomes more active. Structural analysis of the AMPK-AICAR complex suggests that activation of this protein kinase by AICAR mimics activation by AMP [26]. Hence, the effect of AICAR on AMPK is presumed to be direct. 

**Figure 3:**
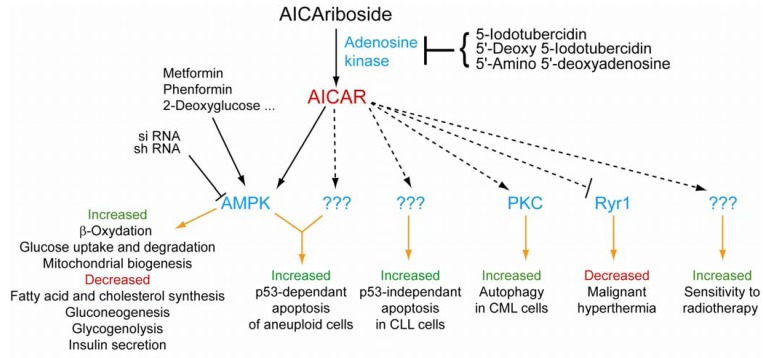
Schematic depiction of AICAR cellular targets and associated biological effects.CML: Chronic myeloid leukemia. CLL: Chronic lymphocytic leukemia. PKC: Protein kinase C. Ryr1: Ryanodine receptor 1. All targets and biological effects presented are described in [29,30,31,32,33,34,35,36].

AICAR monophosphate is provided to the cells as a riboside precursor, which is taken up by adenosine transporter(s) [27]. In many studies, the authors use an inhibitor of adenosine kinase to show that AICAR monophosphate, and not its riboside precursor, is the active molecule (Figure 3) [28]. Among the effects attributed to AICAR monophosphate, many are AMPK-dependent as shown by si- or sh-RNA gene silencing of the gamma-subunits (see Figure 3). For instance the potent effect of AICAR on induction of apoptosis in aneuploid cells was abolished by shRNA on AMPK, however it was poorly mimicked by other AMPK inducers such as metformin or 2-deoxyglucose [29]. This example illustrates the complexity of AICAR effects and calls attention to the fact that a careful analysis is required to establish whether an AICAR effect is fully AMPK-dependent or not.

## 5. AMPK-Independent Effects of AICAR: Other Protein Targets

It should be stressed that there is a growing number of examples where AICAR effects are totally or partially independent of AMPK (Figure 3) [37,38,39,40]. It appears more and more evident that AICAR is a multi-target molecule resulting in complex and combined effects, in line with the paradigm of “network pharmacology” recently proposed by A. Hopkins [41]. For such highly integrated effects, it is critical to apprehend the complexity of the drug effects by identifying its targets. This quest is complex because it requires to identify drug-interacting proteins and establish their role in the drug action *in vivo*. 

The use of an AICAR-resin has allowed validating two yeast transcription factors as AICAR-binders [3]. More recently, affinity chromatography coupled to mass spectrometry allowed us to perform a large-scale identification of yeast and mouse AICAR-binders, many of which being conserved through species (our unpublished results). 

The next step is to validate the binders as true AICAR targets *in vivo*. Interestingly, it was recently found that the yeast AMPK (Snf1) is activated by ADP but not by AMP [42], thereby accounting for the fact that AICAR apparently does not activate the yeast AMPK, as presumed from the transcriptome signature [3]. Yeast is therefore an appealing simple eukaryotic model to study AMPK-independent AICAR effects.

Aside from AMPK, AICAR modulates several enzymes such as glucokinase [43] or glycogen phosphorylase [44]. In a few cases, direct binding of AICAR to specific proteins has been reported, including phosphofructokinase (PFK) and fructose-1,6-biphosphatase (F1,6-BPase) which are inhibited *in vitro* by AICAR [45,46]. AICAR interaction with Hsp90 was also demonstrated and many client proteins of Hsp90 were found destabilized *in vivo* in the presence of AICAR [47]. Both PFK and Hsp90 contribute to important functions for tumor growth and could thus be involved in the anti-proliferative effects of AICAR, which was reported for several tumor cell lines (such as PC-3, MCF-7,C6 glioma, U87MG, K-562 and CEM) [48]. It is noteworthy that, while AICAR replaces AMP in AMPK [26], it competes with ATP in Hsp90 [47]. It will be interesting to determine whether all AICAR targets are nucleotide-binding proteins. 

## 6. Effects of AICAR on Whole Organisms

There are few studies showing effects of AICAR on whole organisms and in most cases the protein effectors are not clearly identified, although AICAR was chosen in these studies for its AMPK-activating properties. 

AICAR feeding of *Caenorhabditis elegans* resulted in decreased fat storage as would be predicted when AMPK is activated [49]. *Drosphila melanogaster* fed with AICAR were more resistant to anoxia/re-oxygenation injuries [50]. AICAR has been found to reduce myocardial ischemic injury in several models (rat, mice, rabbit, dog…) [51] and in humans [52]. Injection of AICAR to mice resulted in a hypoglycemic effect [53]. Strikingly, sedentary mice fed with AICAR showed increased endurance [54]. AICAR was renamed “the exercise pill” and subsequently suspected of human misusage as a doping agent. AICAR is not currently approved by FDA and has only been used in a very few investigations in humans [55,56,57].

## 7. Conclusion

AICAR is a highly promising pharmacophore showing various effects on multiple functions. In the future, AICAR or derivatives could represent key molecules for several diseases including heat induced sudden death, cytochrome c-oxidase deficiencies, cancer and other pathologies associated with muscle wasting. The systematic identification of AICAR targets is now required to understand the complex consequences resulting, most probably, from synthetic effects on several proteins. Importantly, AICAR has both activating and inhibiting effects and hence, determining the way AICAR affects each target will require individual characterization. This time consuming process will hopefully be made easier by the use of model organisms such as yeast or nematode.
